# Standardized Radiomics Analysis of Clinical Myocardial Perfusion Stress SPECT Images to Identify Coronary Artery Calcification

**DOI:** 10.7759/cureus.43343

**Published:** 2023-08-11

**Authors:** Saeed Ashrafinia, Pejman Dalaie, Thomas H Schindler, Martin G Pomper, Arman Rahmim

**Affiliations:** 1 Radiology, Johns Hopkins University School of Medicine, Baltimore, USA; 2 Cardiology, Washington University School of Medicine, St Louis, USA; 3 Physics and Astronomy, University of British Columbia, Vancouver, CAN

**Keywords:** coronary artery calcium score, ai, coronary artery disease, computer aided diagnosis, standardized radiomics, machine learning, cac score, coronary artery calcification, cardiac spect, radiomics

## Abstract

Purpose: Myocardial perfusion (MP) stress single-photon emission computed tomography (SPECT) is an established diagnostic test for patients suspected of coronary artery disease (CAD). Meanwhile, coronary artery calcification (CAC) scoring obtained from diagnostic CT is a highly sensitive test, offering incremental diagnostic information in identifying patients with significant CAD yet normal MP stress SPECT (MPSS) scans. However, after decades of wide utilization of MPSS, CAC is not commonly reimbursed (e.g. by the CMS), nor widely deployed in community settings. We studied the potential of complementary information deduced from the radiomics analysis of normal MPSS scans in predicting the CAC score.

Methods: We collected data from 428 patients with normal (non-ischemic) MPSS (^99m^Tc-sestamibi; consensus reading). A nuclear medicine physician verified iteratively reconstructed images (attenuation-corrected) to be free from fixed perfusion defects and artifactual attenuation. Three-dimensional images were automatically segmented into four regions of interest (ROIs), including myocardium and three vascular segments (left anterior descending [LAD]-left circumference [LCX]-right coronary artery [RCA]). We used our software package, standardized environment for radiomics analysis (SERA), to extract 487 radiomic features in compliance with the image biomarker standardization initiative (IBSI). Isotropic cubic voxels were discretized using fixed bin-number discretization (eight schemes). We first performed blind-to-outcome feature selection focusing on a priori usefulness, dynamic range, and redundancy of features. Subsequently, we performed univariate and multivariate machine learning analyses to predict CAC scores from i) selected radiomic features, ii) 10 clinical features, and iii) combined radiomics + clinical features. Univariate analysis invoked Spearman correlation with Benjamini-Hotchberg false-discovery correction. The multivariate analysis incorporated stepwise linear regression, where we randomly selected a 15% test set and divided the other 85% of data into 70% training and 30% validation sets. Training started from a constant (intercept) model, iteratively adding/removing features (stepwise regression), invoking the Akaike information criterion (AIC) to discourage overfitting. Validation was run similarly, except that the training output model was used as the initial model. We randomized training/validation sets 20 times, selecting the best model using log-likelihood for evaluation in the test set. Assessment in the test set was performed thoroughly by running the entire operation 50 times, subsequently employing Fisher’s method to verify the significance of independent tests.

Results: Unsupervised feature selection significantly reduced 8×487 features to 56. In univariate analysis, no feature survived the false-discovery rate (FDR) to directly correlate with CAC scores. Applying Fisher’s method to the multivariate regression results demonstrated combining radiomics with the clinical features to enhance the significance of the prediction model across all cardiac segments.

Conclusions: Our standardized and statistically robust multivariate analysis demonstrated significant prediction of the CAC score for all cardiac segments when combining MPSS radiomic features with clinical features, suggesting radiomics analysis can add diagnostic or prognostic value to standard MPSS for wide clinical usage.

## Introduction

This paper aims to enhance the clinical utility of routine clinical myocardial perfusion (MP) single-photon emission computed tomography (SPECT) imaging through advanced radiomics analysis. We hypothesize that identifying mild heterogeneities via radiomics analysis can enable the identification of subclinical coronary artery disease (CAD) that would carry important diagnostic and prognostic information. We aim to evaluate our exciting and novel hypothesis that MP SPECT radiomic features extracted from clinically normal (non-ischemic) MP SPECT scans correlate with coronary artery calcification (CAC) as extracted from CT imaging. This section continues with an introduction to MP imaging using SPECT, CAC scoring using CT, and clinical motivations for our work. Subsequently, we describe our methods, followed by results and conclusions. 

Myocardial perfusion stress SPECT

Myocardial perfusion SPECT (MPS) is established for non-invasive evaluations of patients suspected of CAD [1,2]. It is probably the most widely used technique of nuclear cardiology, and its purpose is to assess the adequacy of blood flow to the myocardium [3]. Although MP imaging can be performed with either planar or tomographic techniques [3,4], nowadays tomographic MP imaging through SPECT scanners has become widely popular, more accessible, and more affordable to patients.

MP stress SPECT (MPSS) has an established pathophysiologic basis with radiotracers capturing blood flow. If a patient with CAD is at rest, typically, blood flow through a diseased coronary artery (e.g., narrowed through plaque build-up) is not decreased until coronary stenosis exceeds 90% of the artery. On the other hand, coronary reserve, which refers to the ability to increase coronary blood flow in case of increased metabolic demand, is reduced if coronary stenosis exceeds 50% [5]. As a result, patients who suffered from CAD may have a homogeneous uptake of myocardial blood flow even in the presence of a severely narrowed coronary artery. But the same degree of narrowing can result in reduced flow reserve when the heart is stressed under exercise, resulting in inhomogeneity of regional MP. Such inhomogeneity can be captured using radiotracers that are distributed in the body in proportion to myocardial blood flow [3]. 

CAC quantitation using coronary artery calcium scoring

Large prospective studies have shown that CAC scoring is associated with the risk of future cardiovascular events [6-9]. Studies have shown that noninvasive tests for CAD including electrocardiogram (ECG), ultrasound imaging, and even MP SPECT scan, which are used quite often in cardiac patients’ assessment and diagnosis, were of limited value to detect this calcification due to their low sensitivity [10]. A minimum of 25% of the patients who experience a nonfatal acute myocardial infarction or sudden death do not have previous symptoms [11], and it is necessary to identify asymptomatic individuals at greater risk of future cardiovascular events to plan for preventive strategies.

Agatston is the mainstream CAC scoring method and is often used in clinical practice [12]. The score is calculated for each of the main arteries of the heart, namely the left anterior descending (LAD), the right coronary artery (RCA), and the left circumference (LCX), as well as the left main (LM). This calculation, despite being relatively straightforward, requires special software and the cost associated with its licensing requirements might be another hurdle in the widespread application of CAC scoring in smaller cost-effective radiology centers. 

CAC is a highly specific marker of coronary atherosclerosis, and higher CAC scores are associated with increased plaque burden and increased cardiovascular risk [13,14]. Previous studies demonstrated that a considerable number of stenoses do not result in abnormal perfusion on MP imaging [15,16], which is why in our work we set the inclusion criteria of “non-ischemic normal” MP stress scans. Furthermore, the CAC score is shown to offer incremental diagnostic information over MPS for identifying patients with significant CAD and negative MP imaging results [17]. Therefore, quantifying the uptake heterogeneity from MPSS images aiming at predicting CAC score would be beneficial as it eliminates an additional non-contrast CT for CAC assessment, thus reducing the excessive dose to the patient. Unlike MPS, the CAC test is NOT reimbursed by CMS, while it is known to improve risk stratification in asymptomatic individuals [13,15]; but our study enables CAC assessment from MPSS. Moreover, CAC calculation requires sophisticated software and trained radiologists. Large institutions include this in their CAD patients’ diagnosis package, but is not readily available in community settings.

Utilizing a standardized radiomics framework for reproducibility

Radiomics transforms digitally encrypted medical images that contain information regarding tissue pathophysiology into mineable high-dimensional data [18,19]. In other words, it hypothesizes that different phenotypic characteristics such as intra- and inter-tissue uptake heterogeneity can be quantified as features called “radiomic features” through advanced image processing and computer vision techniques [18]. The information is harnessed through image processing and quantitative image analyses [20] and can be leveraged via clinical decision support systems to improve decision-making and personalized medicine [21]. In this study, we developed a pipeline to evaluate various classes of standardized radiomic features of clinical MPS images. Radiomics is a relatively young discipline and has experienced relatively fast growth, yet, it has not been readily translated to routine clinical practice. This may be due to the low reproducibility of most current studies [22]. Radiomics has a complex workflow involving many steps that often suffers from incomplete reporting of methodologic information. Consequently, few radiomics studies available in the current literature are readily reproducible from start to end [22]. Another major issue is the relatively small number of images in radiomics research datasets that may induce overfitting and high false-positive rates. This further worsens with the tendency to report overly optimistic results [22].

Guidelines and protocols are available for quality control measures in nuclear medicine imaging to standardize patient preparation, dose production, and administration, image acquisition, image reconstruction, standardized uptake value (SUV) normalization, etc., such that the absolute SUV values are interchangeable in multicenter studies [23]. Nonetheless, the methodology to prepare the image and calculate radiomic features is also subject to variability, showing a crucial need for standardization [24-26]. Several studies have shown the importance of robust and standardized protocols to enable reliable quantification of heterogeneity with textural features. They demonstrated an important need to standardize the computation methods due to the complexity of the radiomics workflow [22,24,27]. Since September 2016, an initiative comprising over 50 researchers from 21 top universities and cancer centers, including our group from Johns Hopkins University, has participated in the image biomarker standardization initiative (IBSI) [28]. IBSI aims at standardizing feature computation and image preprocessing phases of the radiomics workflow to ensure its reproducibility.

An IBSI-certified radiomics workflow starts by harmonizing all images within the dataset to ease the variations between different scanners. Features are then calculated based on standardized definitions. Feature selection is performed subsequently, where features are inspected and narrowed down toward improved statistical significance and reduced false-discovery rate (FDR). Finally, by investigating the relationship between radiomic features and the CAC scores, we hope to open a new possibility: to use clinical MPSS scans for additional assessment of CAC. This has important implications, given that, as mentioned above, CAC assessment is not commonly performed nor reimbursed in a wide community setting, and as such, our proposed framework holds promise for new added usage and value for routine MPSS imaging.

Radiomics has witnessed significant activity, especially in oncologic magnetic resonance imaging (MRI), CT, and positron emission tomography (PET). Yet, it has not been thoroughly assessed in 3D SPECT and/or cardiac imaging, partially due to their low spatial resolution that may appear less likely to provide valuable texture and heterogeneity information. However, our group has successfully demonstrated the exciting use of radiomics in brain SPECT [29,30]. At the same time, cardiac SPECT radiomics remains unexplored. Moreover, the prevalence of these scans is significantly higher compared to PET exams, enabling the collection of a higher volume of data for such data-oriented MPS research.

An important ingredient to success in the translation of radiomic features to clinical reality is to quantify and ascertain their robustness, which was one of the aims of this study. The result of our reproducibility analysis would be valuable for future MPS radiomics research by other researchers, as well as for the next steps in our radiomics research toward discovering prognostic cardiac imaging biomarkers. In the following section, we elaborate on our methodology followed by results. We subsequently provide discussions on our findings and a conclusion. This article was previously posted to the medRxiv preprint server on February 01, 2021 [31].

## Materials and methods

**Figure 1:**
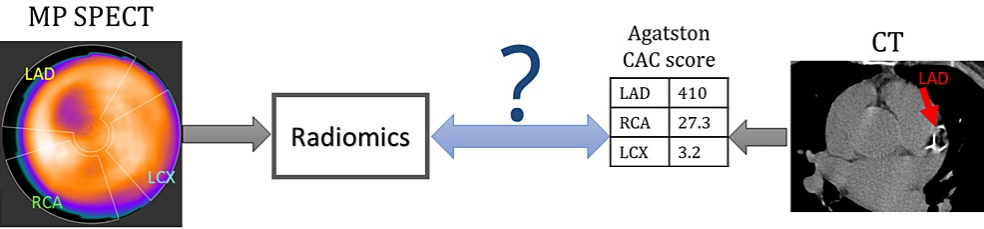
Objective of the study Diagram of the problem addressed in this paper: using radiomics of stress MP stress SPECT to predict CAC scores obtained from CT scan. The CAC scores of main cardiac arteries are calculated using clinical software for LAD, RCA, LCX, and LM. MP, myocardial perfusion; SPECT, single-photon emission computed tomography; CAC, coronary artery calcification; LAD, left anterior descending; RCA, right coronary artery; LCX, left circumflex artery; LM, left main.

Figure 1 depicts the overall aim of this study. We constructed a dataset of 428 clinical MPSS images with a normal scan (non-ischemic) and a separate CT for CAC scoring with their detailed CAC reading.

We constructed a dataset consisting of 428 clinical MPSS images with a normal scan (non-ischemic) with a separate CT for CAC scoring with their detailed CAC reading. We pursue the following three steps:

*Step 1:* Improved quantitative assessment through analysis of standardized radiomic features on MPSS images. We start by identification of patients with normal MPSS tests and CAC CT, followed by image segmentation.

*Step 2*: Eliminating nonreproducible and redundant features (feature selection).

*Step 3*: Use of machine learning techniques to extract CAC information directly from MPSS image radiomics, in contrast to the routine use of CT scans.

Patient collection 

After obtaining approval from the Institutional Review Board (IRB) at Johns Hopkins University, we searched for patients with stress MPS scans from 2011 to 2015. We investigated around 1,800 reports of patients undergoing MPSS, out of which 428 cases were selected. All patients had a CT scan for CAC scoring at the same time as their MPS scan in the PACS database. A nuclear medicine physician (NMP) investigated the MPSS images independent of their CAC scoring report to be free from i) image artifacts, ii) overcorrection, and iii) spillover from nearby liver or stomach. Our NMP also derived detailed CAC scores for each of the arteries of the heart using clinical software.

The dataset consists of images collected from various Siemens®, GE®, and Phillips® hybrid SPECT/CT scanners, at the Johns Hopkins Hospital throughout those years, but all were reconstructed with an “attenuation-corrected iterative reconstruction” (AC-IR) algorithm using the low-dose CT AC acquired at the time of the scan, along with a consistent voxel size of 4.8 mm. According to the quality factors of radiomics research, this is an important characteristic of a study to have imaging acquisition protocols that are “well described and ideally similar across patients,” and “methodologic steps taken to incorporate only images of sufficient quality” [22].

We recorded many parameters for each patient, including basic information (age, gender, race, height, and weight at the scan time), clinical history (smoking, diabetes, hypertension, hyperlipidemia, and family history of cardiac disease), scan info (voxel size, slice thickness), and any possible outcome info.

Image Segmentation 

**Figure 2:**
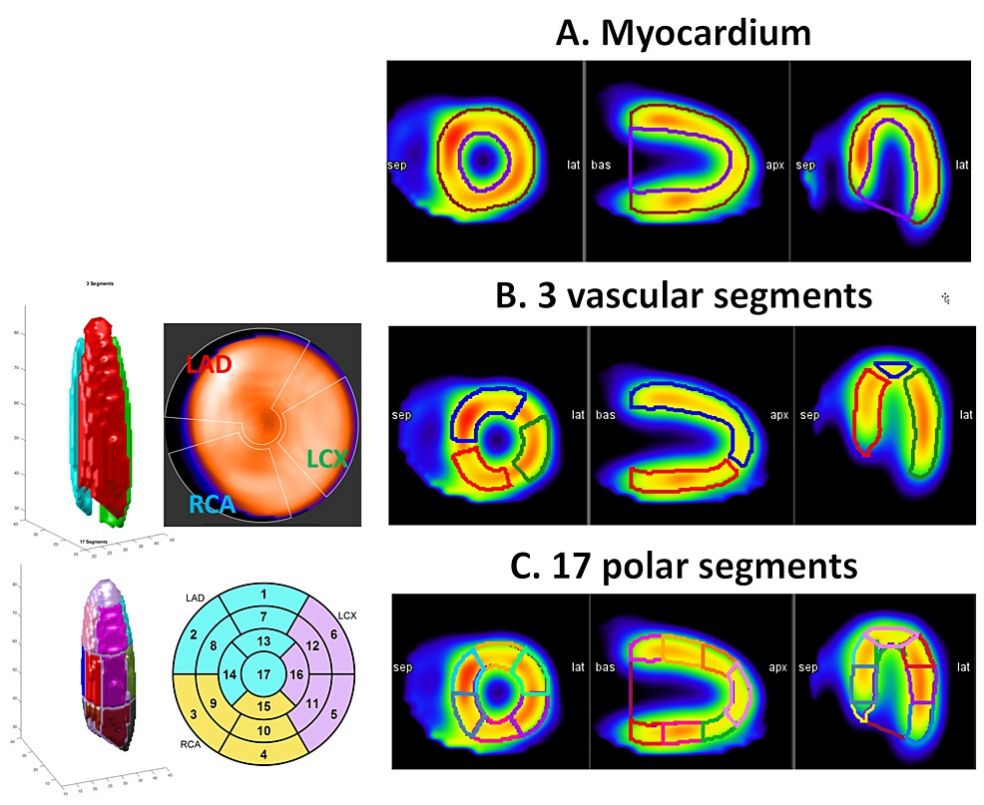
Three cardiac segmentation methods Three methods of segmentation were used in our study. A) The entire myocardium segment. B) Three vascular segments of the heart (LAD, RCA, and LCX), and C) subsets of 17 polar segments of the heart grouped into LAD, LCX, and RCA. LAD, left anterior descending; RCA, right coronary artery; LCX, left circumflex artery.

The study involves three different layers of segmentation as applied to MPSS images: i) the entire myocardium, ii) three vascular segments, and iii) 17 polar segments. Feature evaluation and statistical analysis were performed over all three layers. These three segmentation methods are depicted in Figure 2. The reason we selected two different methods for vascular segmentation is that both methods are widely used in the clinic. The three vascular segment method has a more stringent segment, while the subsets of the 17 polar segments span the whole heart, as can be observed from Figure 2.

We used MIM software^®^ and developed a workflow that automatically draws 3D contours over 21 regions of the heart, namely the endocardium, epicardium, three vascular segments (as depicted in Figure 2B), and 17 polar segments (as depicted in Figure 2C). The workflow was generating the myocardium segment using epi- and endocardium segments. A radiologist supervised this automated segmentation to assure the contours are correctly placed over corresponding segments. Our workflow then exported the 3D SPECT image and these 22 contours as 3D MATLAB^®^ matrices for analysis.

Radiomics framework

We used our in-house-developed standardized environment for radiomics analysis (SERA) package to derive radiomic features that are standardized and reproducible, consistent with the IBSI guideline. IBSI is a global initiative consisting of the world’s top universities and cancer centers [28], in which our group is an active participant [32]. The standardized definition of radiomics terms, features, and feature classes have been well elaborated in [28]. SERA calculates 487 standardized radiomic features aiming to standardize the preprocessing and feature evaluation phases and to meet ISBI’s standards in order to conduct and pursue reproducible research [33]. 

Images produced for MPS scans have arbitrary units - counts - that do not relate to any biological phenomena. Therefore, we ought to use the fixed bin number discretization. We considered and investigated a range of gray level (GL) discretizations, specifically using 4, 8, 16, 32, 64, 128, 256, and 512 bins. All the images in our dataset were reconstructed into 3D images with identical isotropic voxel sizes of $4.8\times 4.8\times 4.8$ mm^3^; thus, no resampling and interpolation were needed. We did not perform any GL rounding or re-segmentation. The framework was then ready to calculate 487 features for eight GLs over seven different segmentations of the heart. 

Statistical analysis

We used statistical analysis to eliminate non-useful features, including features that are identical, nonrobust, and redundant. We performed a multistep feature selection to significantly reduce the size of our feature space of 487×8 features. This process was performed completely independent of the outcome (e.g., CAC score). The selected feature set was subsequently passed on to univariate and multivariate analysis schemes to predict and correlate with clinical outcomes. We also accounted for false discovery by employing Benjamini-Hochberg false-discovery correction method [34].

## Results

Analysis of dataset statistics

**Figure 3:**
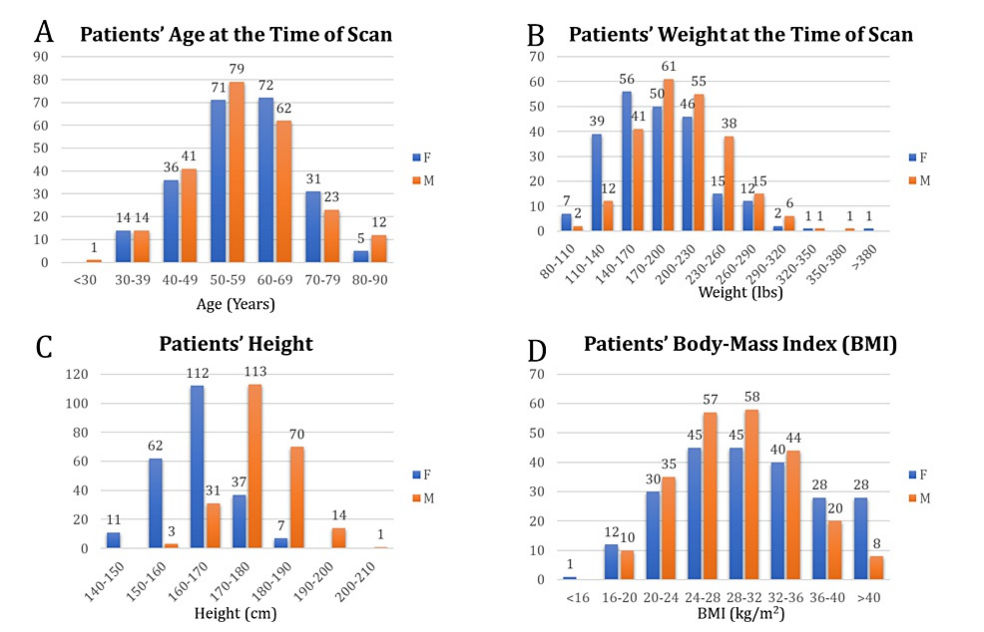
Patients' demographics Distribution of patients’ A) age, B) weight, C) height, and D) BMI at the time of scan grouped into male (orange) and female (blue). BMI, body mass index.

In this section, we present the statistics of data based on the variables previously introduced in the Methods section. The dataset was composed of 229 female (49.7%) and 232 male (50.3%) subjects. Distributions of patient age, height, weight, and body mass index based on gender are depicted in Figure 3.

We observe a relatively close distribution of age between males and females. The frequency of race is presented in Table 1.

**Table 1:** Patients’ race distribution.

	Frequency	Percent
African American	225	48.8
Asian	10	2.2
Hispanic/Latino	7	1.5
Indian	2	0.4
Middle eastern	19	4.1
Native American	1	0.2
White	197	42.7
Total	461	100

Clinical Factors

**Figure 4:**
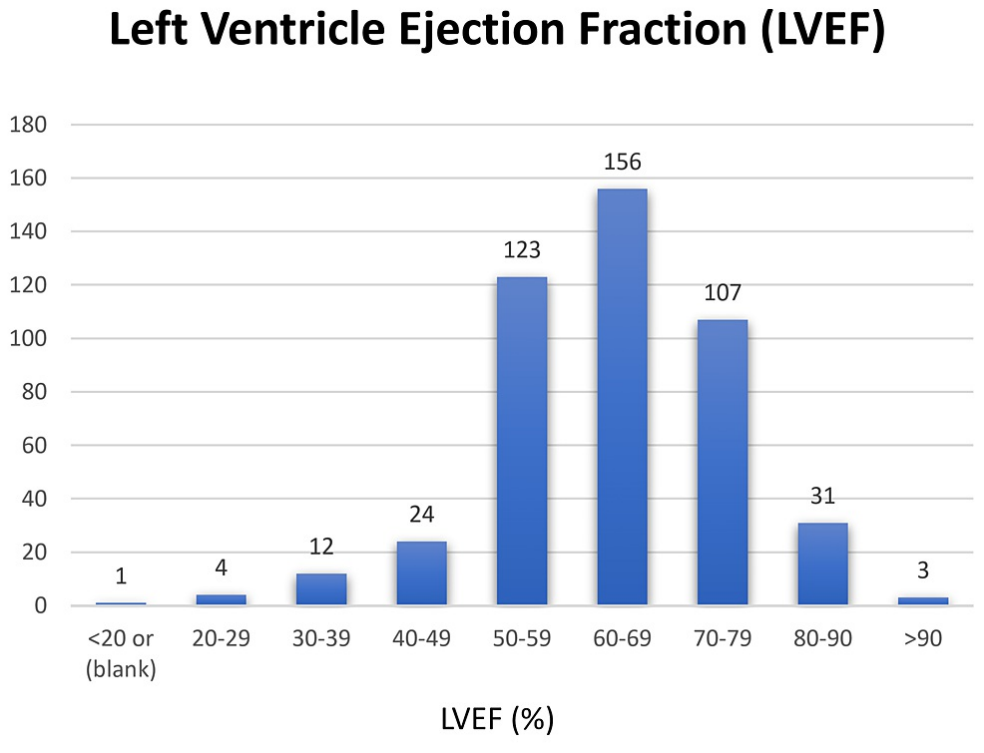
Distribution of LVEF in patients of the dataset LVEF, left ventricle ejection fraction.

Our dataset consists of 428 normal scans. In our dataset, 274 patients received stress compounds by injection (264 A2A adenosine, seven dipyridamole, and two regadenoson), and 186 patients were stressed on a treadmill based on the Bruce protocol [35]. Figure 4 shows the distribution of left ventricular ejection fraction (LVEF) for all patients. The ejection fraction compares the amount of blood in the heart to the amount of blood pumped out. It helps to describe how well the heart is pumping blood to the body. The ejection fraction of a normal heart is between 50% and 70%. A higher LVEF may indicate a heart condition such as hypertrophic cardiomyopathy [36,37]. Other patients’ clinical factors are detailed in Table 2.

**Table 2:** Patients’ clinical factors. CAD, coronary artery disease.

Factor	Attribute	No. of cases	% of Total
Smoking			
	Nonsmoker	220	47.7
	Current smoker	137	29.7
	Previous smoker	104	22.6
Diabetes			
	No	313	67.9
	Yes	148	32.1
Hypertension			
	No	138	29.9
	Yes	323	70.1
Hyperlipidemia			
	No	243	52.7
	Yes	218	47.3
Family history of CAD			
	No	283	61.4
	Yes	178	38.6

**Figure 5:**
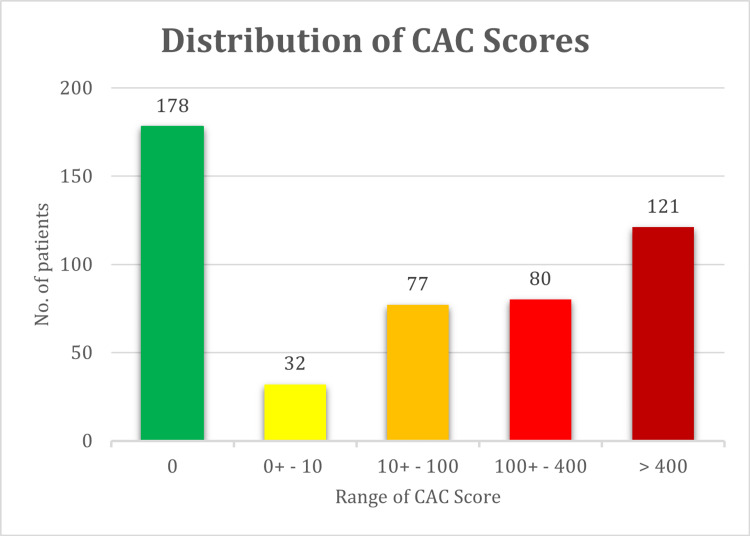
Distribution of patients’ CAC score based on widely used clinical stratification criteria. CAC, coronary artery calcification.

A well-known stratification method presented by Berman et al. divides CAC scores into five established categories 0, 0^+^ to 10, 10^+^ to 100, 100^+^ to 400, 400^+^ to 1000, and >1000 [15]. The distribution of our patients into these five categories is presented in Figure 5.

This shows that 58.4% of the patients with normal MP stress scans had a non-zero CAC score, out of which 33% had a CAC score ≥100. Studies have shown that MP ischemia is rare in patients with CAC <100, whereas in patients with CAC ≥100, the chance of myocardial ischemia increases progressively [15]. Also, one-third of patients with normal MPSS had a CAC score ≥100. As previously mentioned, CAC scoring is known for its high specificity and very small false positives, suggesting that assessment of atherosclerosis burden by CAC scoring may be useful in finding CAD when MPSS fails to report. It also underlies our motivation to develop and study a radiomics-based scheme to extract CAC information directly from MPSS scans.

Feature selection 

SERA calculates all 487 features as defined in the IBSI documentation that are from the 11 main feature categories, namely statistical, morphological, local intensity, histogram, intensity histogram, GL cooccurrence, GL run length, GL size-zone, GL distance-zone, neighboring gray tone difference, neighborhood gray-tone difference, and neighboring gray-level dependence. All these features were initially considered and were calculated for eight GLs.

In this section, we aim to systematically narrow down this large feature set and arrive at a smaller set of meaningful, robust, nonredundant, and reproducible features for further investigation of their predictive or prognostic value, while discouraging overfitting. Our feature selection phase can be generally divided into i) pre-feature calculation and ii) post-feature calculation as explained below. Following feature selection, we discuss how to narrow down to an optimum discretization level.

*Pre-Feature Calculation* 

In the first step, before performing any analysis, we eliminate irrelevant feature families based on the nature of cardiac SPECT images and our knowledge about what each feature captures.

*Removing 2D and 2.5D feature families*: Our dataset originally consists of images with isotropic voxels. Therefore, there would be no additional information provided to us from 2D or 2.5D feature families. These feature families would have been beneficial when slice thickness (i.e. voxel size in z dimension) was different from the voxel size in x and y dimensions. In that case, resizing and interpolating the images to isotropic voxel sizes may have resulted in modification of the original voxel distribution, causing possible loss or modification of data. In any case, the following feature families were eliminated: 2D and 2.5D gray-level co-occurrence matrix (GLCM) (25 features) and gray-level run-length matrix (GLRLM) (16 features) (both merged and averaged), 2D and 2.5D gray-level size zone matrix (GLSZM) (16 features), gray-level distance zone matrix (GLDZM) (16 features), neighborhood gray-tone difference matrix (NGTDM) (five features), and neighboring gray-level dependence matrix (NGLDM) (17 features). This removed 272 features, narrowing down our feature space to 215.

*Removing useless feature families*: MPS images typically have voxels with arbitrary units (they are not quantitative unlike PET or some SPECT imaging applications). Therefore, any feature that conveys information regarding the exact intensity values of the original region of interest (ROI) is not considered meaningful. As such, intensity-based features (18 features) and local intensity features (two features) were excluded. Furthermore, the seven segments were created by an automatic segmentation procedure that generates ROIs with similar shapes (all registered to the same reference space). As such, the shape of the segments does not carry any differentiating information, and we are interested mainly in the heterogeneity caused by voxel intensity variations that carry information about the blood flow in different heart segments. As such, morphological features (29 features) were excluded as well. At the end of this step in our analysis, we were left with 166 features out of 487, eliminating the majority via our knowledge of the underlying nature of the features. 

*Post-Feature Calculation* 

*Removing features with identical values*: Next, we searched for features with identical values across all patients to exclude. In our dataset, four features were found with identical values across all patients: histogram minimum, maximum, and range, and NGLCM dependence count percentage. Once eliminated, we arrived at 162 features. 

**Figure 6:**
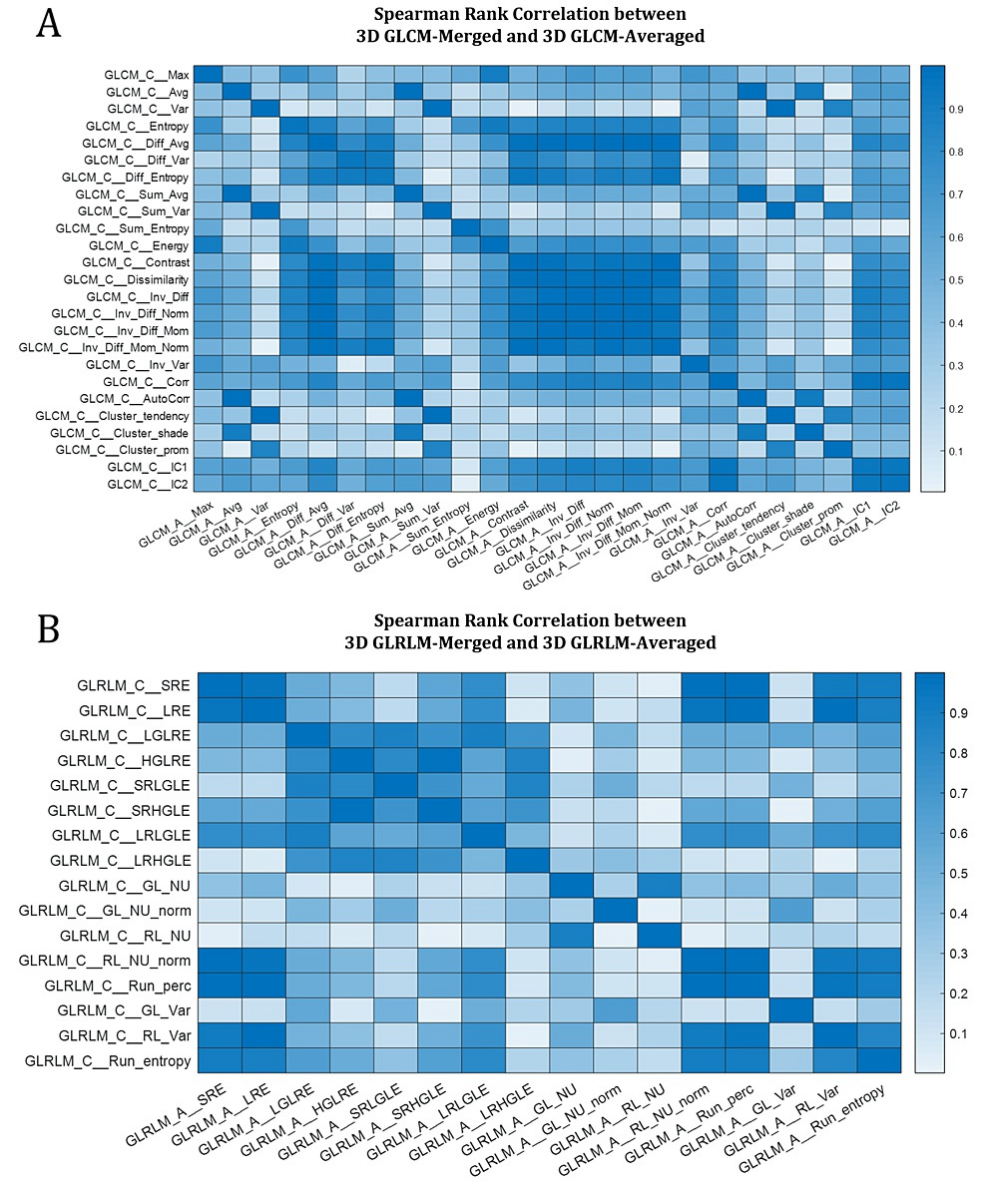
Heatmaps of Spearman rank correlation between A) 3D GLCM-averaged vs. 3D GLCM-merged, and B) 3D GLRLM-averaged vs. 3D GLRLM-merged. Values in the diagonal of both plots are >0.98. The acronyms of the above radiomic features in the GLCM and GLRLM classes are mentioned in [28]. GLCM, gray-level co-occurrence matrix; GLRLM, gray-level run-length matrix.

*Removing feature families with more than one variety*: In the next step, we calculate the Spearman rank correlation between each feature and all other features. This enables us to explore the relationship between features and find redundant and highly correlated features. At this step, every higher-order feature class remained has only one subtype (e.g., only 3D features, after excluding 2D and 2.5D) except for GLCM and GLRLM, and these two classes have two 3D subtypes: 3D merged and 3D averaged. We investigated the correlation between each variety of higher-order 3D calculations (i.e., 3D GLCM averaged vs. merged, and 3D GLRLM averaged vs. merged) and used a systematic approach to narrow them down. Figure 6 shows a heatmap of their correlation. In the diagonal of both heatmaps in Figure 6, we observe a very high Spearman correlation (between 0.98 and 1) between all the same features within the two feature families, i.e., GLCM-averaged entropy vs. 3D GLCM-merged entropy, etc., indicating the redundancy of features calculated in two varieties (merged vs. averaged). Let us consider $S_{\left\{A \right \}\mid \left\{B \right \}}$ as the Spearman rank correlation between feature families $\left \{ A \right \}$ and $\left \{ B \right \}$. We subtracted $S_{\left\{\textrm{3D GLCM-averaged} \right \}\mid \left\{\textrm{All feature families except 3D GLCM-merged} \right \}}$ from $S_{\left\{\textrm{3D GLCM-merged} \right \}\mid \left\{\textrm{All feature families except 3D GLCM-averaged} \right \}}$, and did the same for GLRLM, and observed it yields values very close to zero, which further indicates that using one variety vs. the other does not add additional information to our analysis, suggesting the exclusion of one variety from both GLCM and GLRLM. Subsequently, to decide which of the two varieties to exclude, we calculated the range of features in both varieties and removed the one with a smaller range, which yields to exclusion of the 3D-merged of both categories and keeping 3D GLCM-averaged and 3D GLRLM-averaged. This further reduced the number of features to 121. This observation is also consistent with findings in [38], where the authors reported merged features with tighter distribution in a smaller range and subsequently remove them from the rest of their study.

*Removing redundant features*: After using Spearman correlation to reduce the feature space at the feature-family level, we move on to investigate the correlation at the feature level. The next set of features to remove is the feature pair with a Spearman rank correlation coefficient of 1, indicating their redundancy. These features included three pairs: i) “3D GLSZM-zone percentage (ZP)” and “3D GLDZM-ZP”, ii) “3D GLSZM-GL nonuniformity (NU) normalized” and “3D GLDZM-GL NU normalized”, and iii) “3D GLSZM-GL NU” and “3D GLDZM-GL NU”. From each pair, we selected the feature with a lower range to exclude which yielded the removal of the GLDZM features from each pair. 

*Removing features with a low dynamic range*: In the next step, we calculated the percent variance of the features (variance/mean) representing their dynamic range. Subsequently, we removed features with a very low dynamic range of less than $10^{-5}$, which were five: Histogram-skewness, Histogram-kurtosis, Histogram-min gradient, GLCM-averaged cluster shade, and GLCM-averaged first measure of information correlation. Now the dataset has 113 features. 

*Removing highly correlated features*: In the last step of this phase, using the Spearman correlation of features with respect to each other calculated earlier, we opt to remove highly correlated features as defined by those having a Spearman correlation coefficient $\left | \rho \right |\geq 0.95$ as suggested in the literature [39]. These feature pairs are considered highly correlated and likely to provide redundant rather than complementary information. We remove these features through the following recursive operation.

We use the heatmap of feature-pair Spearman correlation to find features with $\left | \rho \right |\geq 0.95$. We subsequently record the number of instances a feature fits this criterion. Then, we sort these features based on which feature has more instances of $\left | \rho \right |\geq 0.95$ with others in descending order and call it $\boldsymbol{f}^{\boldsymbol{sorted}}$. We then start from the first feature in this set. We denote this first feature by $\boldsymbol{f} ^{\boldsymbol{keep}}$, i.e., the feature to keep, and save it to $\boldsymbol{K}$ the set of features we intend to keep. Subsequently, we mark the highly correlated features with $\boldsymbol{f} ^{\boldsymbol{keep}}$ and save them to an empty set denoted by $\boldsymbol{R}$, i.e., for removal. We then loop over each feature inside $\boldsymbol{R}$ and find other highly correlated features with these features and append them to $\boldsymbol{R}$. Once the procedure is complete, we update $\boldsymbol{f} ^{\boldsymbol{sorted}}$ by removing $\boldsymbol{f} ^{\boldsymbol{keep}}$ and all features inside $\boldsymbol{R}$. The algorithm then continues recursively with this update $\boldsymbol{f} ^{\boldsymbol{sorted}}$, letting its first member be $\boldsymbol{f} ^{\boldsymbol{keep}}$ and append it to $\boldsymbol{K}$, and find features and add them to $\boldsymbol{R}$ for removal. This process continues until $\boldsymbol{f} ^{\boldsymbol{sorted}}$ becomes empty. 

The above algorithm cuts the number of features in half, removing 57 features from 113 and yielding 56 features remaining that are not highly correlated with each other and are more likely to provide complementary information.

Selecting the Best Discretization Level (GLs)

The above procedure reduced the feature set from 487$\times$8 to 56$\times$8 features for eight GLs. Now we focus on discretization levels to systematically remove non-useful GLs. First, we observe that for the three smallest GLs, the number of identical features is higher than for the other five GLs. Furthermore, features with smaller dynamic range increase by 22%, 4%, 29%, and 29% compared to GL =64 or 128. Moreover, the two highest GLs have 11% and 22% more feature pairs with Spearman correlation $\geqslant$ 0.9. Therefore, we can safely remove all GLs except 64 and 128. 

The procedure in the previous paragraph could have been performed without the analysis of the range and Spearman rank correlation of features. We can safely remove the first three GLs since the intervals that voxel intensities were discretized into are so large that they do not provide enough opportunity to capture the heterogeneity of a region. On the other hand, the two largest GLs produce so many bins to discretize voxels into that many bins will be empty or just have very few representations in the ROI. For instance, the LAD segment consists of 460 voxels on average. When it is discretized into 512 GLs, they are more bins than voxels, and many bins would be empty or just occur very scarcely. In this case, our higher-order matrices such as GLRLM, GLSZM, and GLDZM, in which the number of columns represents different run-lengths, zone sizes, distance zones, etc., would be very long and narrow matrices with very small variability. As a result, these higher GLs should be eliminated, too.

Interestingly, this finding is consistent with some previously published studies on the radiomics of PET imaging [38,40].

Finally, out of the remaining two GLs, 64 and 128, we found very similar behavior from both discretization levels in terms of the range of the features and the number of feature pairs with high Spearman correlation. We decided to choose 64 for the rest of this study, because 1) as mentioned GL =128 does not demonstrate different statistical properties, 2) our results in the previous study suggested 64 GLs for the other SPECT study - imaging of renal cell carcinoma with ^99m^Tc, which is the same radiotracer as the one used for MPSS imaging [41], and 3) some previous studies have demonstrated that GL =64 provided higher textural feature reproducibility [42] and robustness [40].

Wrapping Up Feature Selection 

Through the above procedures, we reduced our feature set 487$\times$8 to 56. One important note is that these features were excluded in a *completely unsupervised manner without any involvement in the clinical outcome* (e.g., CAC score, patient survival, etc.). This is a key factor to make our effort statistically sound and more believable. 

Outcome prediction

In this section, we elaborate on efforts toward outcome prediction using the narrowed-down feature set. We also included our negative findings and unsuccessful attempts, as we believe reporting them helps future researchers, and, thus, is of scientific value.

Univariate Analysis

**Figure 7:**
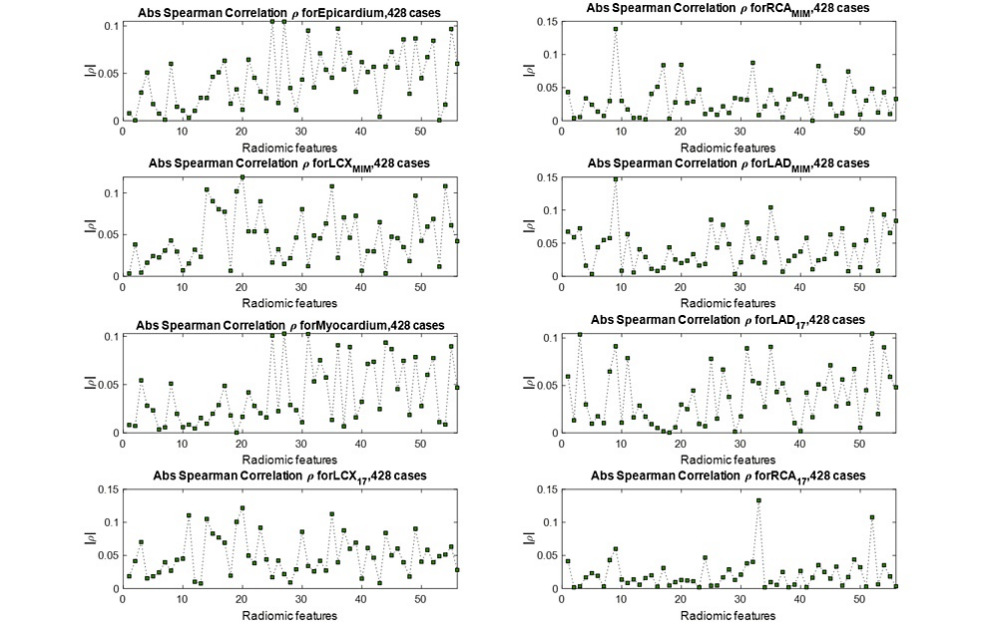
Spearman rank correlation between a selected feature of each segment (56 selected features) and the CAC score of that segment. The maximum correlation observed in all plots is 0.15, which is mediocre. LAD, left anterior descending; RCA, right coronary artery; LCX, left circumflex artery; CAC, coronary artery calcification.

**Figure 8:**
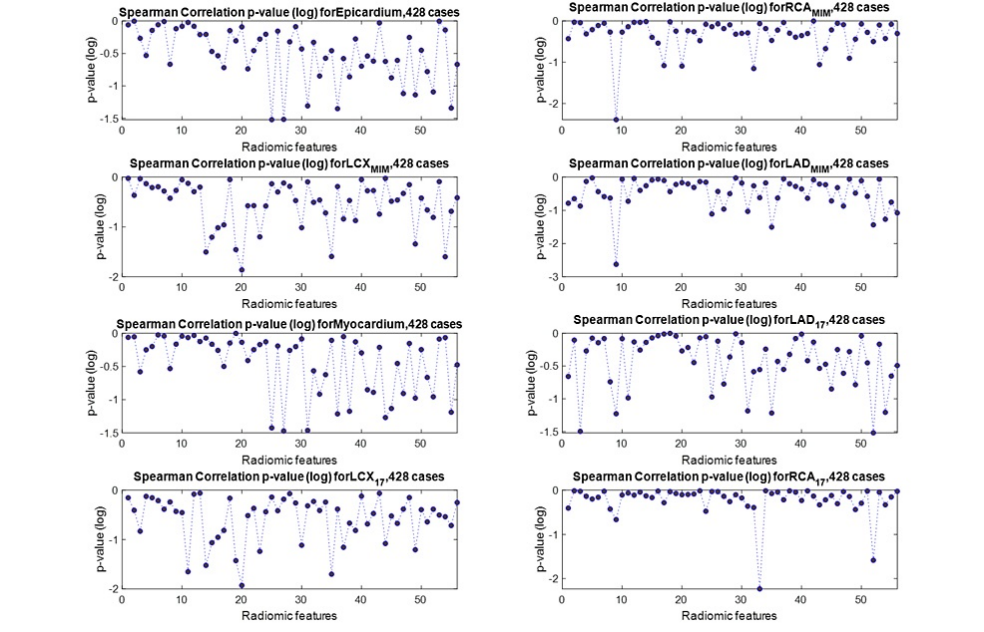
Spearman rank correlation p-value between a selected feature of each segment (56 selected features) and the CAC of that segment. LAD, left anterior descending; RCA, right coronary artery; LCX, left circumflex artery; CAC, coronary artery calcification.

We define our outcome as the CAC score of each region of the heart calculated from the CT scan, and we aim to predict this outcome from the radiomic features extracted from the same region of the MPSS image, as explained in the Methods section. We started by investigating whether our selected radiomic features (previous section) directly correlate with the outcome, that is the CAC score. We adopted two approaches to represent the outcome. In the first approach, the actual CAC score with a continuous scale was utilized. In the second approach, we discretized CAC scores of each region of the heart based on the five-scale clinical stratification criteria explained in the “Clinical factors” section and plotted in Figure 5. Spearman rank correlations between features of every segment with the CAC score of the same segment were calculated for both CAC approaches (continuous and discrete). We also employed the Benjamini-Hochberg FDR correction with $q = 0.05$ to discourage overfitting. None of the features were able to survive FDR correction and still significantly correlate with the outcome under this univariate scheme. Figure 7 shows the absolute value of Spearman correlation coefficient values between 56 selected radiomic features and discretized CAC for eight segments, where we can observe the mediocre correlation values. Figure 8 shows their corresponding p-values (not FDR corrected in this plot). Following the Benjamini-Hochberg FDR correction, no feature survives. This emphasizes the difficulty of the task at hand, and that it is necessary to adopt a more sophisticated, multivariate algorithm for regression (for continuous CAC outcome) or classification (for discrete CAC outcome).

Multivariate Analysis

We observed from Figure 7 that in general, the correlation values between features and CAC scores are relatively low. Despite their low correlation, these selected features had a relatively higher significance, suggesting that while none of these slightly significant features are highly correlated with the outcome, a certain multivariate combination of them might be predictive and provide significant prediction information. Thus, we pursue a multivariate approach to predict CAC scores. In this subsection, first, we introduce stepwise linear regression, then we describe how we handle feature selection. We then explain how our proposed algorithm manages data to produce a fair analysis, and finally, we run the analysis for three different configurations: i) radiomics features only, ii) clinical features only, and iii) radiomic + clinical features and present the results.

*Stepwise linear regression*: In this stage, we pursue a multivariate analysis approach employing stepwise linear regression. Stepwise regression is a systematic method for adding and removing terms from a linear or generalized linear model based on their statistical significance in explaining the response variable. The method begins with an initial model, which in our case is a linear model, and then compares the explanatory power of incrementally larger and smaller models, which is performed by adding or removing terms by stepwise regression and returning the linear model at the end. The initial fit can be a linear or a constant (intersect) model. After the initial fit, the function examines a set of available features and adds the best one to the model if an F-test for adding the term results in a *p*-value of $P_{enter}$, or less. If no terms can be added, it examines the terms currently in the model and removes the worst one if an F-test for removing it has a *p*-value of $P_{remove}$, or greater. This process is repeated until no terms can be added or removed. The constant term (intercept) is never removed from the model. 

*Feature handling*: At each step, the method searches for terms to add to or remove from the model based on a criterion, which we set to be the Akaike information criterion (AIC), a commonly used estimator of the relative quality of statistical models for a given dataset. The AIC method estimates the quality of each model relative to other models, providing a mean for model selection. It reduces the chance of overfitting and underfitting by providing a balance between the goodness of fit and having too many parameters [43].

We can specify the order this algorithm starts to add features and later removes them. Instead of an unstructured approach of starting from the arbitrary first feature in the list, we developed a feature selection method to enter those with higher Spearman correlation to the model first. For this purpose, the Spearman rank correlation between each individual feature in the training set and the outcome (CAC score of the same segment) was calculated. The Spearman correlation coefficients and their corresponding *p*-values were recorded. Then, merely significant features with a *p*-value smaller than a certain range (e.g. 0.3) were selected and others were discarded. The selected features were then sorted into descending order, based on the value of their Spearman correlation. The input dataset is then rearranged based on this subset of Spearman correlation-sorted features to enter features with the highest correlation to the stepwise algorithm first.

*Training/cross-validation/testing setup*: The following procedures were performed for each of the cardiac segments separately. First, the given dataset was shuffled and 15% of the data was set aside as the “independent test set.” This set was not used until the very end of an independent assessment. Then, the following procedure was performed 20 times: the remaining 85% of the data “training + dev set” was randomly divided into training and cross-validation sets with 75%/25% ratios. The procedure described in the previous subsection has already reduced the number of radiomic features to 56. We use the procedure described in the previous subsection to further reduce the number of features and input more useful features for the regression algorithm first. We subsequently perform stepwise linear regression on the training set. We set $P_{enter}$ as 0.05 and $P_{remove}$ as 0.2. 

Once the training is over, we perform cross-validation using the dev set. Cross-validation aims to reduce overfitting to the training set. The cross-validation algorithm is configured the same as training, except for the training algorithm the initial fit was a constant (intercept), whereas for cross-validation the initial fit is the output fit from the training dataset. During the above steps, we recorded the model, including the set of features remaining in it, the value of the log-likelihood, the *p*-value, and the final AIC.

The model fit is typically composed of several features that survived the stepwise algorithm, and it might be possible that only the intercept term survives. If by coincidence the best model consists of only the intercept term, we skip that and choose the best fit with more than one term. 

Following the above procedure, we select the model with the highest AIC of the 20 runs to run on the independent test set blind to the entire operation. To assess its prediction performance, Pearson’s correlation was used to assess the relationship between the two distributions (prediction vs. actual) and subsequently recorded the correlation coefficients ($\rho$) and their corresponding *p*-values. The above operation was performed for each of the segmented lesions of the heart separately. 

**Figure 9:**
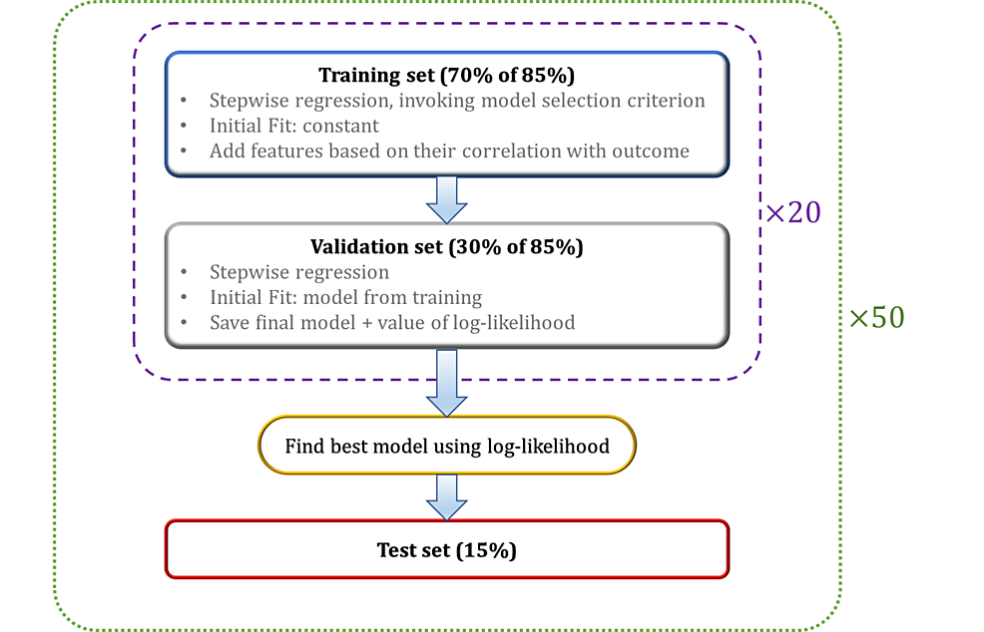
A flowchart of the proposed machine-learning classification algorithm

This is not where we come to a conclusion yet. We kept the test set aside during the whole analysis to assure a completely independent and blind-to-training assessment; however, our result might still be biased to a specific randomly selected test set chosen. To mitigate such a bias even further, we took an extra step and run the entire above operation 50 times. That is, randomly shuffling and dividing the dataset into “training + dev” and “test” sets 50 times, then running the stepwise algorithm 20 times over the “training + dev” set. We subsequently perform 50 predictions on 50 independent test sets that give us the 50 best regression fits and their *p*-values, which we subsequently used to derive our conclusion. A flowchart of the algorithm is depicted in Figure 9.

*Running the multivariate analysis for three configurations*: We performed the above entire operation three times: A) with radiomic features only (imaging), B) with clinical features (non-imaging), and C) with both radiomics and clinical features. The 10 clinical features employed were i) gender, ii) race, iii) age, iv) smoking, v) diabetes, vi) hypertension, vii) hyperlipidemia, viii) family history of cardiac disease, ix) BMI, and x) LVEF. We also assured that a certain subset of clinical features such as gender, race, diabetes, etc. was treated as “categorical” variables, as opposed to continuous, by the algorithm. 

Analysis results

We used Fisher’s method for combining *p*­-values and use the chi-squared distribution test to determine if the stepwise regression method yields a significant fit after running 50 independent trials [44]. Based on this method, under the null hypothesis, the statistics of Fisher’s method computed as

$$X= -2\sum_{i=1}^{N}\log \left (p_i \right )$$

follows a chi-squared distribution with a degree of freedom of $2\times N$, where N is the total number of runs (in our case, $N=50$). Comparing the value of $X$ to the appropriate chi-squared distribution can determine whether the sample is significant. Assuming a significant level of 0.01, the value of the chi-squared distribution for a degree of freedom of $2\times 50=100$ is 135.81. Table 3 shows the result of applying Fisher’s method to the three configurations, where significant results are shown in bold. We observed that radiomic features were unable to yield a significant model for any of the segmentation, and clinical features were able to result in a significant fit for most of the segments. But the combined clinical + radiomic features result in a significant fit across all segments. 

**Table 3:** The value of the chi-squared distribution for each segment and feature configuration. The value of the chi-squared distribution with a degree of freedom of 100 is 135.81, and values above this threshold (shown in bold) are considered significant under the null hypothesis. MIM shows the segmentation performed using three vascular segments (LAD, RCA, and LCX) using MIM software, while 17 shows segmentation according to standard 17 polar segments. LAD, left anterior descending; RCA, right coronary artery; LCX, left circumflex artery.

	RCA_MIM_	LCX_MIM_	LAD_MIM_	Myocardium	LAD_17_	LCX_17_	RCA_17_
Radiomics	95.87	88.02	115.02	111.93	139.25	53.8	53.28
Clinical	84.12	153.14	253.13	294.43	253.13	153.14	84.12
Combined	174.53	194.73	348.97	341.39	326.97	189.2	141.6

**Figure 10:**
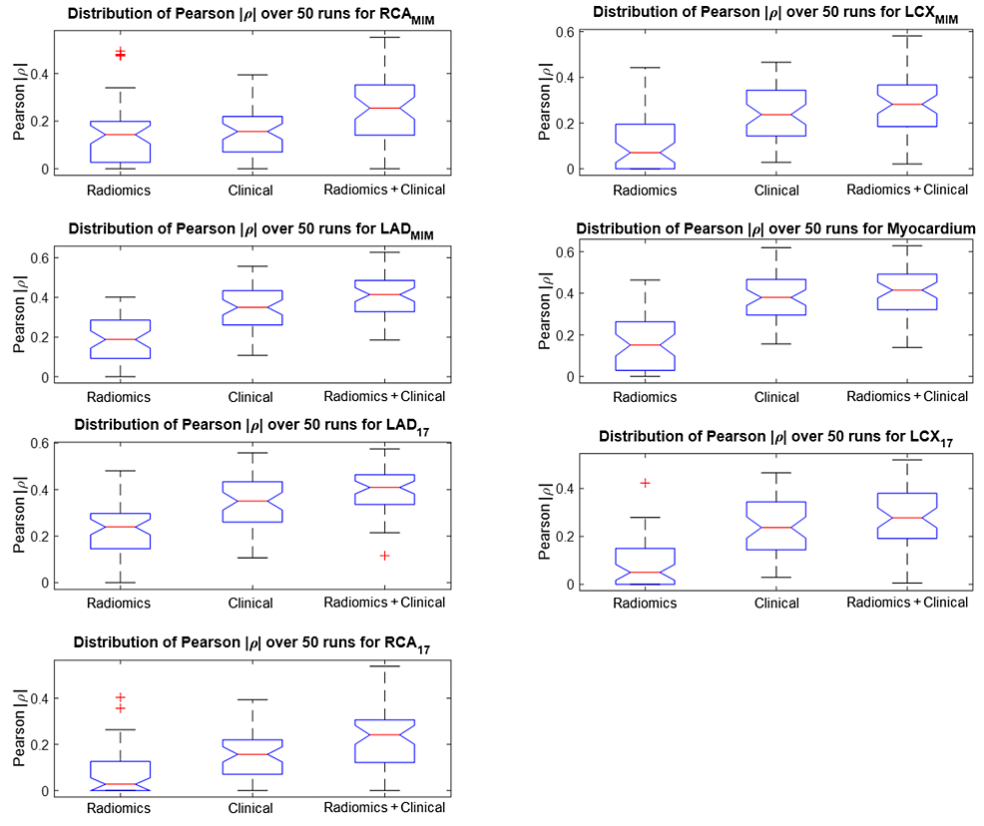
Pearson correlation coefficient results for each cardiac segment Distribution of absolute values of the Pearson’s coefficient of the best fit out of 50 randomized trials of stepwise linear regression for radiomics, clinical and combined features, and for all seven segmentations as defined in the Methods section (the higher, the better). Adding radiomics to clinical features increases the correlation to the CAC score of the corresponding ROI. LAD, left anterior descending; RCA, right coronary artery; LCX, left circumflex artery; ROI, region of interest; CAC, coronary artery calcification.

**Figure 11:**
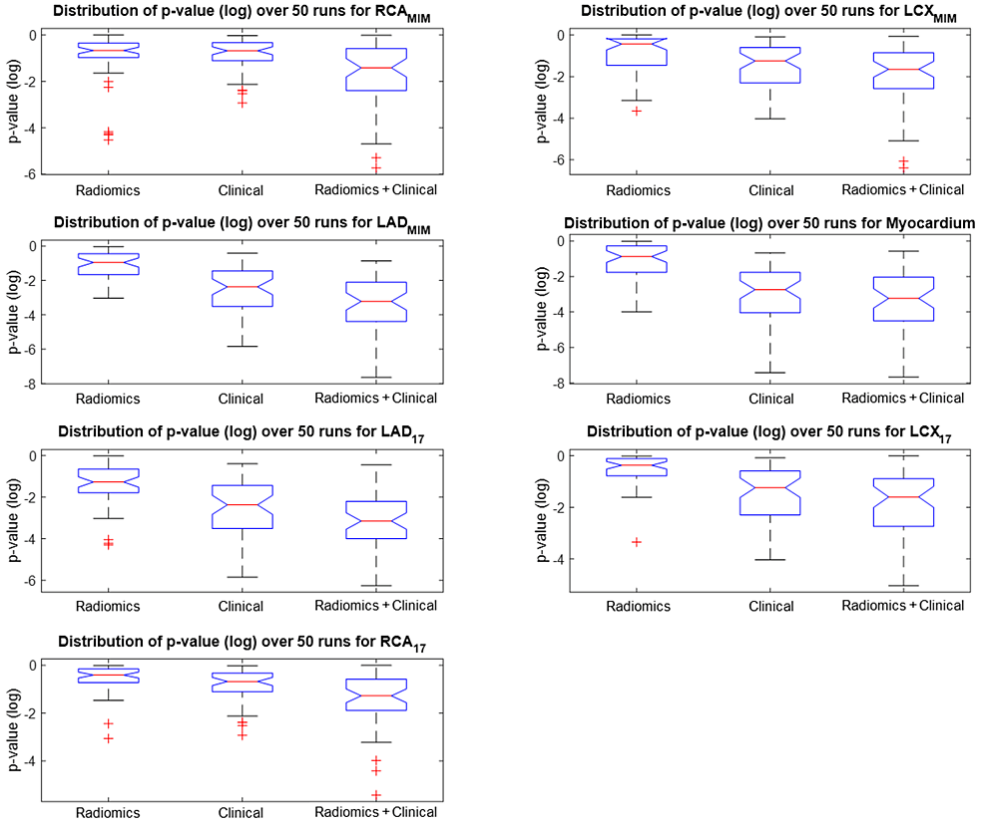
Distribution of p-values (log-scale) of the best fit out of 50 randomized trials Distribution of *p*-values (log-scale) of the best fit out of 50 randomized trials of stepwise linear regression for radiomics, clinical and combined features, and for all seven segmentations as defined in the Methods section (the lower, the better). Adding radiomics to clinical features is seen to enhance the regression significance across all segmentations. LAD, left anterior descending; RCA, right coronary artery; LCX, left circumflex artery.

Figure 10 shows the distribution of the absolute value of Pearson’s correlation coefficient $\left | \rho \right |$ for all seven segments. We observe the same pattern across all segments that the combined radiomics + clinical features are more correlated to the CAC scores of that region. Moreover, Figure 11 shows the distribution of *p*-values of the best fit out of the 50 independent runs of the stepwise regression algorithm, each including 20 model fits where the best is selected. This plot shows that adding radiomics to the 10 clinical features will enhance the significance of the regression model and promise a more robust prediction. 

In clinical configurations, the most prevalent features in the best fit were somehow consistent across different segmentations and included age, hyperlipidemia, hypertension, and smoking. In the combined configuration, usually, the age variable was the first in the fit, followed by hyperlipidemia, GLSZM-small zone large GL emphasis, and GLDZM-short distance large GL emphasis. 

## Discussion

The current proposal is the first demonstration of employing radiomics of normal MPSS to predict CAC score as derived from the CT scan. To our knowledge, no study has been published on the radiomics of cardiac SPECT imaging. Moreover, we did not find any study with the same approach as ours that incorporates readily reconstructed 3D images and preserves the voxel intensities. They focus on using the polar plot for their analyses, which is a 2D projection of the 3D reconstructed image. Recently, few studies have investigated the use of deep learning to predict CAD [45,46]; nonetheless, no studies, to our knowledge, exist on predicting CAC scores from the 3D images of SPECT scans, which is, as indicated earlier, a very challenging task.

Challenges with the proposed idea

The study of MPSS radiomics is a challenging task due to several reasons. First, SPECT is a low-resolution imaging modality that results in a substantial loss of heterogeneity information that had the potential to provide extra knowledge about the blood flow and other functionalities of the heart that could have been captured by radiomics. Moreover, the lack of quantitation in SPECT imaging further causes a major loss of information, resulting in a mostly qualitative interpretation of the scan. Of course, the absence of quantitation prevents the utilization of many useful radiomic features. It also impedes performing cross-scan comparisons. Another drawback of nonquantitative SPECT images can be explained by an example of a patient who has calcification in all three main arteries but has a uniform uptake in his SPECT image reported as normal. This can be due to a condition where blood flow is reduced in all three main arteries, resulting in uniformly decreased flow all around the heart. But since blood flow is not quantifiable, this effect cannot be noticed. However, methods to perform quantitative SPECT scans have been published and even recently been commercialized [47,48]. Quantitative SPECT is shown to carry many clinical implications [49] and promises an increased chance of more accurate and impactful radiomics analysis of the heart.

**Figure 12:**
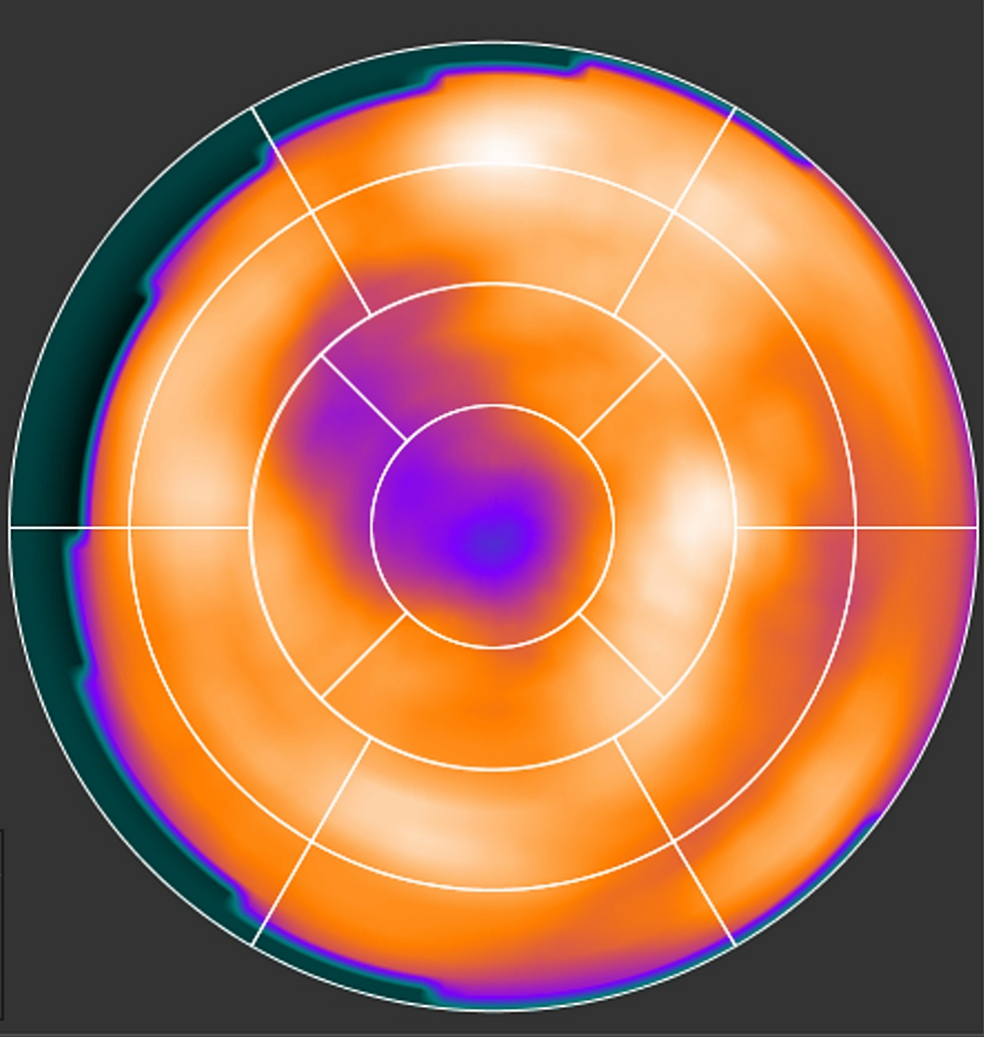
A normal MPSS with apical thinning. MPSS, myocardial perfusion stress SPECT.

Another reason that significantly contributes to the challenges in SPECT radiomics is heterogeneity caused by inherent artifacts of SPECT imaging. MPSS, specifically, can cause artifacts on the reconstructed image that can appear as reduced uptake in the image. An example of this is shown in Figure 12. This effect is called apical thinning and is a well-known phenomenon in MPS. It is often attributed to a reduced myocardial thickness at the apex of the left ventricle. Attenuation correction during the reconstruction appears to exaggerate this effect [50]. Moreover, soft tissue attenuation artifacts also impact MPS images [51]. These artifacts generally appear as fixed defects. Attenuation due to breast tissue usually results in a perfusion defect along the anterior wall of the left ventricle, also affecting the lateral wall, septum, and apex [52]. The effect would be similar to that in Figure 12. During our data collection phase, we observed many cases with this effect apparent in their reconstructed image. Undoubtedly, the heterogeneity caused by this effect may be captured by the radiomic features, while it is completely irrelevant to calcifications in arteries.

**Figure 13:**
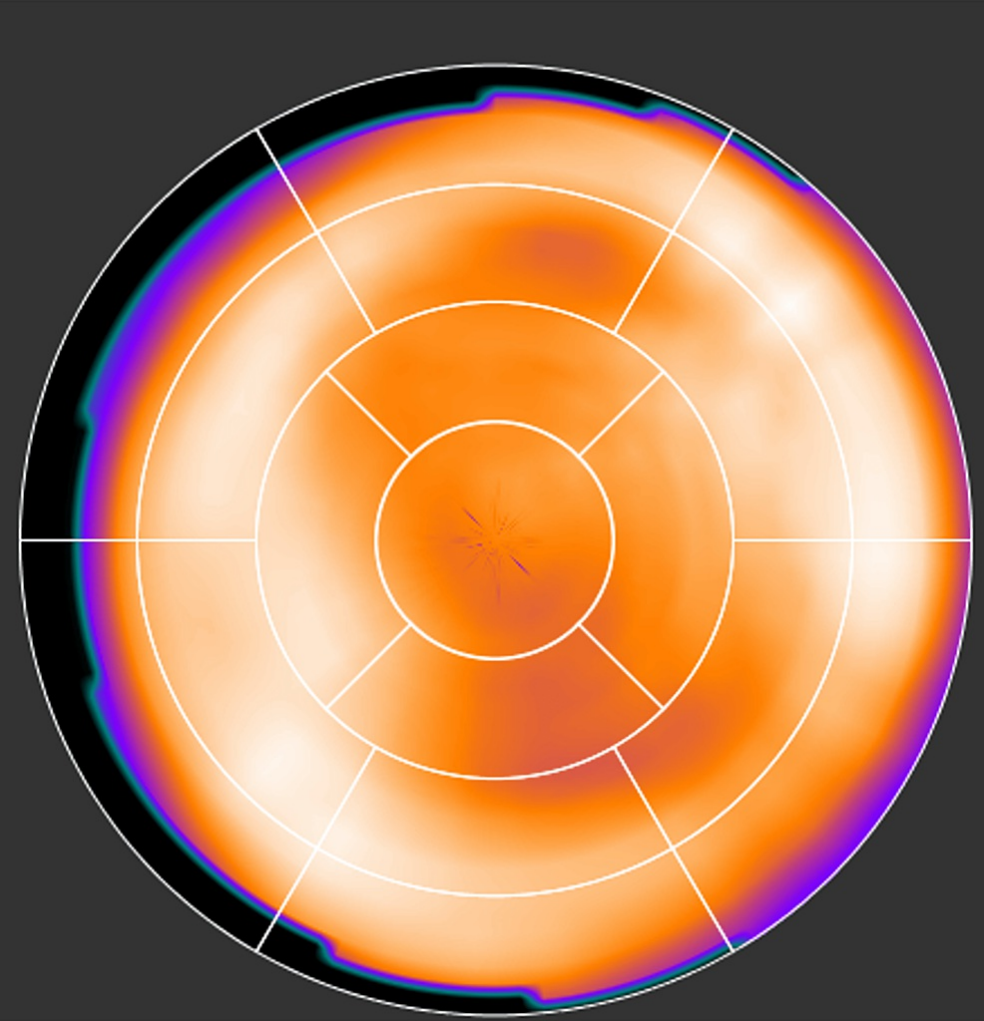
A normal MPSS with severe calcification. This scan is reported as normal due to relatively uniform uptake with no reversibility and/or fixed defect, but the CAC CT scan shows an extraordinary CAC score of 2239. Our proposed research promises to assist in finding such cases with elevated CAC scores. MPSS, myocardial perfusion stress SPECT; CAC, coronary artery calcification.

Figure 13 shows an example of an MPSS scan image in a polar plot form, which is a 2D projection of the 3D SPECT image into its apex (center circle). This image is interpreted as normal, due to the absence of any reversibility and/or defect. But the CT scan of this patient shows an enormous calcification in the arteries of this patient, having an outstanding CAC score of 2239.

Intuition from radiomics

We mentioned that radiomic features mostly included in the fit were GLSZM GLSZM-small zone large GL emphasis and GLDZM-short distance large GL emphasis. Both features emphasize higher GLs, and higher GLs in a discretized SPECT image depict higher blood flow. It is interesting to observe and seems intuitive that the radiomic features that capture higher blood flow in each cardiac segment end up being in the fit. 

Our other efforts 

We wish to also point out that we explored more than 10 other regression methods, including different types of regression trees, support vector machine (SVM) regressors, etc., as well as several classification techniques (bagging, SVM, K-nearest neighbor, etc.) to find a significant prediction model, but our investigation did not return any significant results from these techniques. Yet, we do not exclude the possibility that with further tuning, those algorithms can potentially return significant results.

The significance of the study

In the current study, after many feature elimination steps discussed in the feature selection section, and significantly reducing the feature space by a factor of 70, univariate analysis was not able to find any potential correlation with the outcome. On the other hand, our multivariate analysis carefully designed to mitigate the impact of dataset bias on the outcome prediction was able to successfully predict all segments of the heart. Our statistical analysis in the above section showed that just around 60% of the patients had a non-zero CAC score and one-third of them had a CAC score$\geqslant$100 that is shown to progressively increase the chance of myocardial ischemia. As a result, our multivariate analysis has the potential to make a prediction of CAC which is the most prevalent type of atherosclerosis, showing promise for this study. 

## Conclusions

This study investigated the hypothesis that heterogeneity in MPSS images can possibly convey information regarding calcification in coronary arteries. Many community settings are incapable of providing a CAC CT scan for patients. It is not reimbursed by the CMS and requires sophisticated software. We employed our in-house-developed standardized package, SERA, which was proven to evaluate 487 radiomic features according to IBSI's guidelines. We segmented MPSS images into LAD, RCA, and LCX with two methods, in addition to the whole myocardium, to evaluate radiomic features for all seven segments. We also explored eight GLs to find the most appropriate setting for our study that yields higher reproducibility, robustness, and less redundancy. Our dataset consists of 428 patients with normal (non-ischemic) MPSS images that were verified to be free from artifact or spillover and their detailed CAC scores acquired from CT and other clinical parameters. We focused on patients with normal stress scans since the possible prediction of CAC in those images would have been of clinical significance.

Through a multi-step blind-to-outcome unsupervised feature selection phase, we significantly reduced the feature space 70-fold from 487×8 to 56 features. We also performed the entire operation 50 times to randomly divide our dataset into “training + dev” and “test” sets to mitigate any bias to a specific set of test data. Our univariate analysis using the Spearman rank correlation between each feature of the cardiac segment with the corresponding CAC score of that segment was not significant. Our multivariate analysis, however, was able to significantly predict the CAC score of all cardiac segments when combining radiomic features with clinical features. Our method has the potential to identify such cases with high CAC that can be prompted for more appropriate care, suggesting that radiomics analysis adds diagnostic and prognostic value to standard MPS for wide clinical usage.

## Figures and Tables

**Figure 14:**
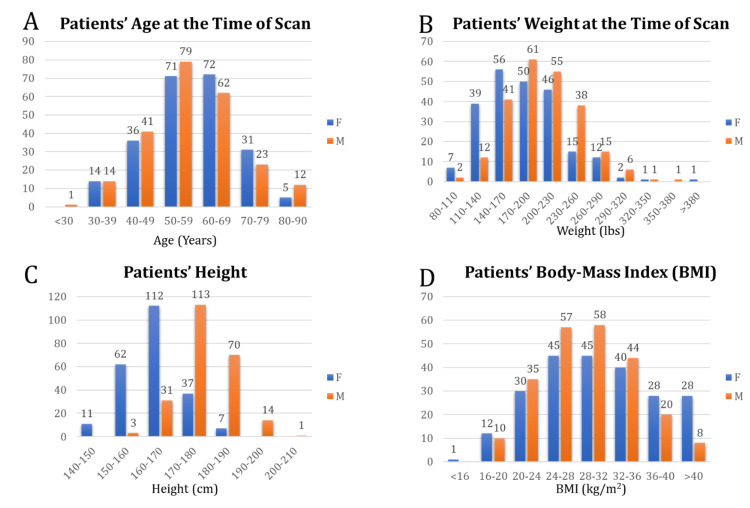
Distribution of A) age, B) weight, C) height, and D) body mass index (BMI) at the time of scan grouped into male (orange) and female (blue)

**Figure 15:**
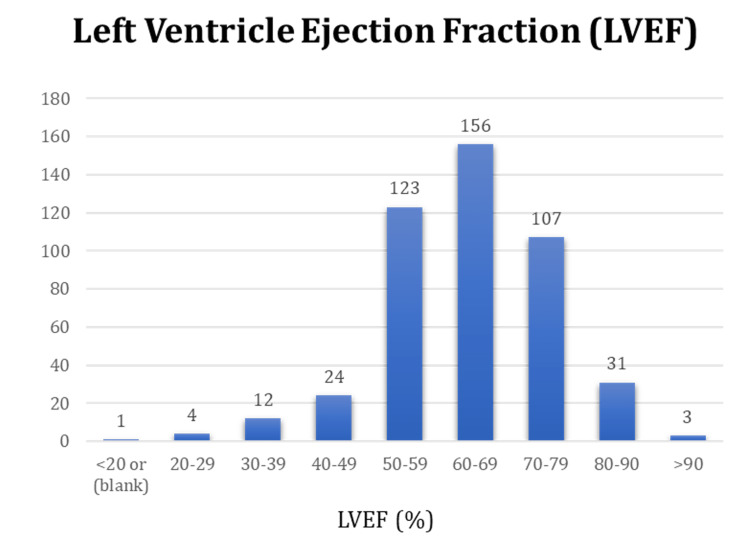
Distribution of left ventricle ejection fraction (LVEF) in patients of our dataset

## Appendices

Additional statistics on patients in this study

Figure 14 shows additional patient statistics, including distributions of patients' age, weight, height, and their body mass index (BMI).

Figure 15 shows the distribution of patients' left ventricle ejection fraction (LVEF) in our cohort. 

Standardized environment for radiomics analysis (SERA)

Introduction

The SERA is a MatLab®-based framework developed at Johns Hopkins University that calculates radiomic features based on guidelines from the Image Biomarker Standardization Initiative (IBSI) (https://arxiv.org/pdf/1612.07003.pdf). SERA is capable of processing images from various clinical imaging modalities such as CT, MRI, PET, and SPECT. Radiomic features calculated with SERA are standardized and in compliance with IBSI, which ensures their reproducibility. The standardized definition of radiomics terms, features, and feature classes have been well elaborated in [28].

Radiomic Features 

SERA calculates 487 IBSI-standardized features (as outlined in Table 4). These include 79 first-order features (morphology, statistical, histogram, and intensity-histogram features), 272 higher-order 2D features, and 136 3D features. Different subsets of features can be selected, such as the default of 215 features (first-order + higher-order 3D). 

**Table 4:** Radiomic features SERA calculates in each feature family. Different 2D, 2.5D, and 3D configurations are explained in detail in the IBSI guideline. Users can set to return only a selected subset of these features. Standardized Environment for Radiomics Analysis (SERA) is our standardized in-house-developed radiomics framework.

Feature family	Subtypes	Number of features
Morphology	-	29
Local intensity	-	2
Intensity-based statistics	-	18
Intensity histogram	-	23
Intensity-volume histogram	-	7
Gray level co-occurrence matrix (GLCM)	2D Averaged	25
Gray level co-occurrence matrix (GLCM)	2D Slice-merged	25
Gray level co-occurrence matrix (GLCM)	2.5D Direction merged	25
Gray level co-occurrence matrix (GLCM)	2.5D All merged	25
Gray level co-occurrence matrix (GLCM)	3D Averaged	25
Gray level co-occurrence matrix (GLCM)	3D Merged	25
Gray level run length matrix (GLRLM)	2D Averaged	16
Gray level run length matrix (GLRLM)	2D Slice-merged	16
Gray level run length matrix (GLRLM)	2.5D Direction merged	16
Gray level run length matrix (GLRLM)	2.5D All merged	16
Gray level run length matrix (GLRLM)	3D Averaged	16
Gray level run length matrix (GLRLM)	3D Merged	16
Gray level size zone matrix (GLZSM)	2D	16
Gray level size zone matrix (GLZSM)	2.5D	16
Gray level size zone matrix (GLZSM)	3D	16
Gray level distance zone matrix (GLDZM)	2D	16
Gray level distance zone matrix (GLDZM)	2.5D	16
Gray level distance zone matrix (GLDZM)	3D	16
Neighborhood gray tone difference matrix (NGTDM)	2D	5
Neighborhood gray tone difference matrix (NGTDM)	2.5D	5
Neighborhood gray tone difference matrix (NGTDM)	3D	5
Neighboring gray level dependence matrix (NGLDM)	2D	17
Neighboring gray level dependence matrix (NGLDM)	2.5D	17
Neighboring gray level dependence matrix (NGLDM)	3D	17
Total		487

Feature Evaluation Settings

SERA has options to set and modify all parameters defined or used in the IBSI guideline. The following parameters can be set up in the image preparation setting of SERA (for detailed information please refer to IBSI documentation [1]):

Resampling and interpolation: resample to 2D and 3D isotropic voxel sizes; interpolation algorithm used in resampling image and ROI (nearest/linear/cubic); partial volume threshold (mostly used for CT HU).

Discretization: bin size, discretization type (fixed bin size/fixed bin numbers), discretization algorithm (uniform/Lloyd).

Other: gray-level rounding, image re-segmentation (range re-segmentation, outliers’ re-segmentation). 

Radiomic Feature Values for IBSI Benchmark Datasets as Calculated by SERA

IBSI shared two phantoms between all participating institutions in two phases' ROIs to facilitate the process of establishing reference values for features. In phase I, it was a small 80-voxel three-dimensional digital phantom with a 74-voxel ROI mask to facilitate the process of establishing reference values for features, without involving image processing. In phase II, a publicly available CT image of a patient with lung cancer with an accompanying gross tumor volume as the ROI [1,2]. We have included a supplemental spreadsheet containing the features calculated by SERA on the IBSI benchmark CT phantom in the Appendix. Table 5 contains the values of each feature computed by SERA in comparison to the IBSI-reported benchmark values for Configuration D of the CT phantom as indicated in the IBSI guideline [1], which is the closest configuration to the setup in the current study. The results in this spreadsheet demonstrate the compliance of SERA with IBSI radiomics guidelines. 

**Table 5:** List of radiomics features calculated by SERA for Configuration D of IBSI CT phantom. SERA shows great compliance with IBSI with 99.8% match in calculated results. Refer to [28] for the standardized definitions of radiomic features and acronyms based on the Image Biomarker Standardization Initiative (IBSI) guidelines. Standardized Environment for Radiomics Analysis (SERA) is our standardized in-house-developed radiomics framework.

Family	Image_Biomarker	Benchmark Value	Tolerance	SERA Result	Diff	Check
Morphology	Volume (mesh-based)	367000	6000	367453.66	0	match
Morphology	Volume (counting)	368000	6000	367880	0	match
Morphology	Surface area	34300	400	34306.254	0	match
Morphology	Surface to volume ratio	0.0934	0.0007	0.093	0	match
Morphology	Compactness 1	0.0326	0.0002	0.033	0	match
Morphology	Compactness 2	0.378	0.004	0.378	0	match
Morphology	Spherical disproportion	1.38	0.01	1.383	0	match
Morphology	Sphericity	0.723	0.003	0.723	0	match
Morphology	Asphericity	0.383	0.004	0.383	0	match
Morphology	Centre of mass shift	64.9	2.8	64.926	0	match
Morphology	Maximum 3D diameter	125	1	125.06	0	match
Morphology	Major axis length	93.3	0.5	93.27	0	match
Morphology	Minor axis length	82	0.5	82.005	0	match
Morphology	Least axis length	70.9	0.4	70.902	0	match
Morphology	Elongation	0.879	0.001	0.879	0	match
Morphology	Flatness	0.76	0.001	0.76	0	match
Morphology	Volume density (AABB)	0.478	0.003	0.478	0	match
Morphology	Area density (AABB)	0.678	0.003	0.678	0	match
Morphology	Volume density (OMBB)	N. A.	N. A.	0.526		N. A.
Morphology	Area density (OMBB)	N. A.	N. A.	0.723		N. A.
Morphology	Volume density (AEE)	1.29	0.01	1.294	0	match
Morphology	Area density (AEE)	1.62	0.01	1.605	0.01	match
Morphology	Volume density (MVEE)	N. A.	N. A.	0.615		N. A.
Morphology	Area density (MVEE)	N. A.	N. A.	1.121		N. A.
Morphology	Volume density (convex hull)	0.834	0.002	0.834	0	match
Morphology	Area density (convex hull)	1.13	0.01	1.13	0	match
Morphology	Integrated intensity	-8640000	1560000	-8641752	0	match
Morphology	Moran's I index	0.0622	0.0013	0.062	0	match
Morphology	Geary's C measure	0.851	0.001	0.851	0	match
Local intensity	Local intensity peak	201	10	200.821	0	match
Local intensity	Global intensity peak	N. A.	N. A.	200.821		N. A.
Statistics	Mean	-23.5	3.9	-23.518	0	match
Statistics	Variance	32800	2100	32786.871	0	match
Statistics	Skewness	-2.28	0.06	-2.28	0	match
Statistics	(Excess) kurtosis	4.35	0.32	4.351	0	match
Statistics	Median	42	0.4	42	0	match
Statistics	Minimum	-724	12	-724	0	match
Statistics	10th percentile	-304	20	-304	0	match
Statistics	90th percentile	86	0.1	86	0	match
Statistics	Maximum	521	22	521	0	match
Statistics	Interquartile range	57	4.1	57	0	match
Statistics	Range	1240	40	1245	0	match
Statistics	Mean absolute deviation	123	6	122.543	0	match
Statistics	Robust mean absolute deviation	46.8	3.6	46.827	0	match
Statistics	Median absolute deviation	94.7	3.8	94.73	0	match
Statistics	Coefficient of variation	-7.7	1.01	-7.699	0	match
Statistics	Quartile coefficient of dispersion	0.74	0.011	0.74	0	match
Statistics	Energy	1.48e9	1.4e9	1.48e9	0	match
Statistics	Root mean square	183	7	182.592	0	match
Intensity histogram	Mean	18.5	0.5	18.503	0	match
Intensity histogram	Variance	21.7	0.4	21.69	0	match
Intensity histogram	Skewness	-2.27	0.06	-2.268	0	match
Intensity histogram	Kurtosis	4.31	0.32	4.308	0	match
Intensity histogram	Median	20	0.5	20	0	match
Intensity histogram	Minimum	1	0	1	0	match
Intensity histogram	10th percentile	11	0.7	11	0	match
Intensity histogram	90th percentile	21	0.5	21	0	match
Intensity histogram	Maximum	32	0	32	0	match
Intensity histogram	Mode	20	0.4	20	0	match
Intensity histogram	Interquartile range	2	0.06	2	0	match
Intensity histogram	Range	31	0	31	0	match
Intensity histogram	Mean absolute deviation	3.15	0.05	3.151	0	match
Intensity histogram	Robust mean absolute deviation	1.33	0.06	1.328	0	match
Intensity histogram	Median absolute deviation	2.41	0.04	2.407	0	match
Intensity histogram	Coefficient of variation	0.252	0.006	0.252	0	match
Intensity histogram	Quartile coefficient of dispersion	0.05	0.0021	0.05	0	match
Intensity histogram	Entropy	2.94	0.01	2.94	0	match
Intensity histogram	Uniformity	0.229	0.003	0.229	0	match
Intensity histogram	Maximum histogram gradient	7260	200	7263	0	match
Intensity histogram	Maximum gradient gray level	19	0.4	19	0	match
Intensity histogram	Minimum histogram gradient	-6670	230	-6674	0	match
Intensity histogram	Minimum gradient gray level	22	0.4	22	0	match
Intensity vol histogram	Volume fraction at 10% intensity	0.972	0.003	0.972	0	match
Intensity vol histogram	Volume fraction at 90% intensity	0.00009	0.000415	0	0	match
Intensity vol histogram	Intensity at 10% volume	87	0.1	87	0	match
Intensity vol histogram	Intensity at 90% volume	-303	20	-303	0	match
Intensity vol histogram	Volume fraction diff between 10% and 90% intensity	0.971	0.001	0.971	0	match
Intensity vol histogram	Intensity difference between 10% and 90% volume	390	20	390	0	match
Intensity vol histogram	Area under the IVH curve	0.563	0.012	0.563	0	match
GLCM (3D, averaged)	Joint maximum	0.232	0.007	0.232	0	match
GLCM (3D, averaged)	Joint average	18.9	0.5	18.852	0	match
GLCM (3D, averaged)	Joint variance	17.6	0.4	17.628	0	match
GLCM (3D, averaged)	Joint entropy	4.95	0.03	4.947	0	match
GLCM (3D, averaged)	Difference average	1.29	0.01	1.293	0	match
GLCM (3D, averaged)	Difference variance	5.37	0.11	5.369	0	match
GLCM (3D, averaged)	Difference entropy	2.13	0.01	2.134	0	match
GLCM (3D, averaged)	Sum average	37.7	0.8	37.705	0	match
GLCM (3D, averaged)	Sum variance	63.4	1.3	63.441	0	match
GLCM (3D, averaged)	Sum entropy	3.68	0.02	3.676	0	match
GLCM (3D, averaged)	Angular second moment	0.11	0.003	0.11	0	match
GLCM (3D, averaged)	Contrast	7.07	0.13	7.071	0	match
GLCM (3D, averaged)	Dissimilarity	1.29	0.01	1.293	0	match
GLCM (3D, averaged)	Inverse difference	0.682	0.003	0.682	0	match
GLCM (3D, averaged)	Inverse difference normalized	0.965	0.001	0.965	0	match
GLCM (3D, averaged)	Inverse difference moment	0.656	0.003	0.656	0	match
GLCM (3D, averaged)	Inverse difference moment normalized	0.994	0.001	0.994	0	match
GLCM (3D, averaged)	Inverse variance	0.341	0.005	0.341	0	match
GLCM (3D, averaged)	Correlation	0.798	0.005	0.798	0	match
GLCM (3D, averaged)	Autocorrelation	370	16	369.511	0	match
GLCM (3D, averaged)	Cluster tendency	63.4	1.3	63.441	0	match
GLCM (3D, averaged)	Cluster shade	-1270	40	-1272.93	0	match
GLCM (3D, averaged)	Cluster prominence	35700	1400	35664.719	0	match
GLCM (3D, averaged)	Information correlation 1	-0.231	0.003	-0.231	0	match
GLCM (3D, averaged)	Information correlation 2	0.845	0.003	0.845	0	match
GLCM (3D, merged)	Joint maximum	0.232	0.007	0.232	0	match
GLCM (3D, merged)	Joint average	18.9	0.5	18.852	0	match
GLCM (3D, merged)	Joint variance	17.6	0.4	17.638	0	match
GLCM (3D, merged)	Joint entropy	4.96	0.03	4.965	0	match
GLCM (3D, merged)	Difference average	1.29	0.01	1.29	0	match
GLCM (3D, merged)	Difference variance	5.38	0.11	5.381	0	match
GLCM (3D, merged)	Difference entropy	2.14	0.01	2.139	0	match
GLCM (3D, merged)	Sum average	37.7	0.8	37.703	0	match
GLCM (3D, merged)	Sum variance	63.5	1.3	63.506	0	match
GLCM (3D, merged)	Sum entropy	3.68	0.02	3.679	0	match
GLCM (3D, merged)	Angular second moment	0.109	0.003	0.109	0	match
GLCM (3D, merged)	Contrast	7.05	0.13	7.045	0	match
GLCM (3D, merged)	Dissimilarity	1.29	0.01	1.29	0	match
GLCM (3D, merged)	Inverse difference	0.682	0.003	0.682	0	match
GLCM (3D, merged)	Inverse difference normalized	0.965	0.001	0.965	0	match
GLCM (3D, merged)	Inverse difference moment	0.657	0.003	0.657	0	match
GLCM (3D, merged)	Inverse difference moment normalized	0.994	0.001	0.994	0	match
GLCM (3D, merged)	Inverse variance	0.34	0.005	0.34	0	match
GLCM (3D, merged)	Correlation	0.8	0.005	0.8	0	match
GLCM (3D, merged)	Autocorrelation	370	16	369.503	0	match
GLCM (3D, merged)	Cluster tendency	63.5	1.3	63.506	0	match
GLCM (3D, merged)	Cluster shade	-1280	40	-1275.262	0	match
GLCM (3D, merged)	Cluster prominence	35700	1500	35742.844	0	match
GLCM (3D, merged)	Information correlation 1	-0.225	0.003	-0.225	0	match
GLCM (3D, merged)	Information correlation 2	0.846	0.003	0.846	0	match
GLRLM (3D, averaged)	Short runs emphasis	0.734	0.001	0.734	0	match
GLRLM (3D, averaged)	Long runs emphasis	6.66	0.18	6.657	0	match
GLRLM (3D, averaged)	Low GL run emphasis	0.0257	0.0012	0.026	0	match
GLRLM (3D, averaged)	High GL run emphasis	326	17	325.741	0	match
GLRLM (3D, averaged)	Short run low GL emphasis	0.0232	0.001	0.023	0	match
GLRLM (3D, averaged)	Short run high GL emphasis	219	13	218.622	0	match
GLRLM (3D, averaged)	Long run low GL emphasis	0.0484	0.0031	0.048	0	match
GLRLM (3D, averaged)	Long run high GL emphasis	2670	30	2667.075	0	match
GLRLM (3D, averaged)	GL non-uniformity	3290	10	3293.649	0	match
GLRLM (3D, averaged)	GL non-uniformity normalized	0.133	0.002	0.133	0	match
GLRLM (3D, averaged)	Run length non-uniformity	12400	200	12351.102	0	match
GLRLM (3D, averaged)	Run length non-uniformity normalized	0.5	0.001	0.5	0	match
GLRLM (3D, averaged)	Run percentage	0.554	0.005	0.554	0	match
GLRLM (3D, averaged)	GL variance	31.5	0.4	31.453	0	match
GLRLM (3D, averaged)	Run length variance	3.35	0.14	3.348	0	match
GLRLM (3D, averaged)	Run entropy	5.08	0.02	5.081	0	match
GLRLM (3D, merged)	Short runs emphasis	0.736	0.001	0.736	0	match
GLRLM (3D, merged)	Long runs emphasis	6.56	0.18	6.556	0	match
GLRLM (3D, merged)	Low GL run emphasis	0.0257	0.0012	0.026	0	match
GLRLM (3D, merged)	High GL run emphasis	326	17	326.073	0	match
GLRLM (3D, merged)	Short run low GL emphasis	0.0232	0.001	0.023	0	match
GLRLM (3D, merged)	Short run high GL emphasis	219	13	219.402	0	match
GLRLM (3D, merged)	Long run low GL emphasis	0.0478	0.0031	0.048	0	match
GLRLM (3D, merged)	Long run high GL emphasis	2630	30	2625.593	0	match
GLRLM (3D, merged)	GL non-uniformity	42800	200	42767.969	0	match
GLRLM (3D, merged)	GL non-uniformity normalized	0.134	0.002	0.134	0	match
GLRLM (3D, merged)	Run length non-uniformity	160000	3000	160418.5	0	match
GLRLM (3D, merged)	Run length non-uniformity normalized	0.501	0.001	0.501	0	match
GLRLM (3D, merged)	Run percentage	0.554	0.005	7.199	6.66	no match
GLRLM (3D, merged)	GL variance	31.4	0.4	31.425	0	match
GLRLM (3D, merged)	Run length variance	3.29	0.13	3.295	0	match
GLRLM (3D, merged)	Run entropy	5.08	0.02	5.083	0	match
GLZSM(3D)	Small zone emphasis	0.637	0.005	0.637	0	match
GLZSM(3D)	Large zone emphasis	99100	2800	99078.516	0	match
GLZSM(3D)	Low GL emphasis	0.0409	0.0005	0.041	0	match
GLZSM(3D)	High GL emphasis	188	10	188.183	0	match
GLZSM(3D)	Small zone low GL emphasis	0.0248	0.0004	0.025	0	match
GLZSM(3D)	Small zone high GL emphasis	117	7	116.553	0	match
GLZSM(3D)	Large zone low GL emphasis	241	14	240.778	0	match
GLZSM(3D)	Large zone high GL emphasis	41400000	300000	41404348	0	match
GLZSM(3D)	GL non-uniformity	212	6	212.134	0	match
GLZSM(3D)	GL non uniformity normalized	0.0491	0.0008	0.049	0	match
GLZSM(3D)	Zone size non-uniformity	1630	10	1629.113	0	match
GLZSM(3D)	Zone size non-uniformity normalized	0.377	0.006	0.377	0	match
GLZSM(3D)	Zone percentage	0.0972	0.0007	0.097	0	match
GLZSM(3D)	GL variance	32.7	1.6	32.718	0	match
GLZSM(3D)	Zone size variance	99000	2800	98972.773	0	match
GLZSM(3D)	Zone size entropy	6.52	0.01	6.515	0	match
GLDZM(3D)	Small distance emphasis	0.579	0.004	0.579	0	match
GLDZM(3D)	Large distance emphasis	10.3	0.1	10.258	0	match
GLDZM(3D)	Low GL emphasis	0.0409	0.0005	0.041	0	match
GLDZM(3D)	High GL emphasis	188	10	188.183	0	match
GLDZM(3D)	Small distance low GL emphasis	0.0302	0.0006	0.03	0	match
GLDZM(3D)	Small distance high GL emphasis	99.3	5.1	99.3	0	match
GLDZM(3D)	Large distance low GL emphasis	0.183	0.004	0.183	0	match
GLDZM(3D)	Large distance high GL emphasis	2620	110	2619.168	0	match
GLDZM(3D)	GL non-uniformity	212	6	212.134	0	match
GLDZM(3D)	GL non-uniformity normalized	0.0491	0.0008	0.049	0	match
GLDZM(3D)	Zone distance non-uniformity	1370	20	1369.445	0	match
GLDZM(3D)	Zone distance non-uniformity normalized	0.317	0.004	0.317	0	match
GLDZM(3D)	Zone percentage	0.0972	0.0007	0.097	0	match
GLDZM(3D)	GL variance	32.7	1.6	32.718	0	match
GLDZM(3D)	Zone distance variance	4.61	0.04	4.614	0	match
GLDZM(3D)	Zone distance entropy	6.61	0.03	6.614	0	match
NGTDM (3D)	Coarseness	0.000208	0.000004	0	0	match
NGTDM (3D)	Contrast	0.046	0.0005	0.046	0	match
NGTDM (3D)	Busyness	5.14	0.14	5.144	0	match
NGTDM (3D)	Complexity	400	5	399.694	0	match
NGTDM (3D)	Strength	0.162	0.008	0.162	0	match
NGLDM(3D)	Low dependence emphasis	0.0912	0.0007	0.091	0	match
NGLDM(3D)	High dependence emphasis	223	5	222.748	0	match
NGLDM(3D)	Low GL count emphasis	0.0168	0.0009	0.017	0	match
NGLDM(3D)	High GL count emphasis	364	16	364.049	0	match
NGLDM(3D)	Low dependence low GL emphasis	0.00357	0.00004	0.004	0	match
NGLDM(3D)	Low dependence high GL emphasis	18.9	1.1	18.945	0	match
NGLDM(3D)	High dependence low GL emphasis	0.798	0.072	0.798	0	match
NGLDM(3D)	High dependence high GL emphasis	92800	1300	92761.625	0	match
NGLDM(3D)	GL non-uniformity	10200	300	10172.049	0	match
NGLDM(3D)	GL non-uniformity normalized	0.229	0.003	0.229	0	match
NGLDM(3D)	Dependence count non-uniformity	1840	30	1836.865	0	match
NGLDM(3D)	Dependence count non-uniformity normalized	0.0413	0.0003	0.041	0	match
NGLDM(3D)	Dependence count percentage	1	0	1	0	match
NGLDM(3D)	GL variance	21.7	0.4	21.69	0	match
NGLDM(3D)	Dependence count variance	63.9	1.3	63.923	0	match
NGLDM(3D)	Dependence count entropy	6.98	0.01	6.981	0	match
NGLDM(3D)	Dependence count energy	0.0113	0.0002	0.011	0	match

Radiomics quality factors

Table 6 contains a cross-check list of radiomics quality factors, including details on how our study has implemented every factor [3,53]. 

**Table 6:** Radiomics quality factor implemented in the current study AIC is Akaike Information Criterion. MPSS is Myocardial Perfusion Stress SPECT. Refer to [28] for the standardized definitions of radiomic features and acronyms based on the Image Biomarker Standardization Initiative (IBSI) guidelines. Standardized Environment for Radiomics Analysis (SERA) is our standardized in-house-developed radiomics framework.

	Radiomics quality factor	Considered	Comment
Imaging	Standardized imaging protocols	Yes	A standardized protocol was in place across all Johns Hopkins Hospital SPECT scanners
	Imaging quality insurance	Yes	Scanners were well maintained and calibrated to produce high-quality clinical images
	Calibration	Yes	Image post-processing and standardized radiomics calculation with SERA
Experimental setup	Multi-institutional/external datasets	No	Left for future studies…
	Prospective study	No	Left for future studies…
Feature selection	Feature robustness	Yes	Feature robustness was studied against segmentation variations, discretization schemes, etc. It was performed independent of the outcome.
	Feature complementarity	Yes	Extensively implemented, reducing 487 to 56 features.
Model assessment	False-discovery correction	Yes	Implementing Benjamini-Hochberg for univariate, AIC, and Fisher’s methods for multivariate.
	Estimation of model performance	Yes	The training model was further optimized on the validation set.
	Independent testing	Yes	The independent test set was blind to training/validation. Feature selection was blind to outcome.
	Performance results consistency	Yes	The consistency of results was demonstrated via 50 times randomly shuffling the {training-validation}/test sets.
	Comparison to conventional metrics	Yes	Radiomics results were compared against conventional (clinical) metrics.
	Multivariable analysis with non-radiomic variables	Yes	Multivariate analysis of combined radiomics + clinical features were provided.
Clinical implications	Biological correlate	Yes	We provide intuition explaining why specific radiomics features (e.g. GLSZM-small zone large GL emphasis) appear in the multivariate model fit.
	Potential clinical application	Yes	Potential clinical application is to predict/stratify patients’ CAC score based on the radiomics of MPSS.
Material availability	Open data	No	Not available
	Open code	Yes	Provided in the Appendix section
	Open models	Yes	The result of our independent-to-outcome feature selection process has been provided for future studies in the radiomics of MPSS.
